# Ten simple rules for starting research in your late teens

**DOI:** 10.1371/journal.pcbi.1008403

**Published:** 2020-11-19

**Authors:** Cameron Mura, Mike Chalupa, Abigail M. Newbury, Jack Chalupa, Philip E. Bourne

**Affiliations:** 1 Department of Biomedical Engineering, University of Virginia, Charlottesville, Virginia, United States of America; 2 School of Data Science, University of Virginia, Charlottesville, Virginia, United States of America; 3 City Neighbors Foundation, Baltimore, Maryland, United States of America; Carnegie Mellon University, UNITED STATES

## Introduction

The Ten Simple Rules (TSR) series covers topics ranging from the very broad (e.g., career paths and scientific communication) to the more specific (e.g., illustrating figures and managing software), and all the various TSRs focus on one’s scientific and professional development [1]. The present TSR shares that goal and is authored by consumers and suppliers of research opportunities. Here, the consumers are individuals in their late teenage years, i.e., late high school (LHS) or early college (EC), who are either considering or actively searching for their first opportunity in a research lab at a university, national lab, or beyond (authors AMN and JC). The suppliers are university researchers (CM and PEB), along with the views of a seasoned educator (MC). We write this TSR for 3 reasons. First, research requests have become more frequent in recent years and, while our general area has been computational biology, that’s probably secondary: The Rules articulated here may apply equally well across many disciplines. Second, in the past decade or so, there has been an astonishing increase in the *intensity* of HS students, on many fronts—in terms of technical skill sets (e.g., mastery of programming languages), academic preparation and scientific sophistication (e.g., courses in advanced math), and beyond (e.g., career-related ambitions, such as searching for research opportunities!). Finally, some of these HS students who spent time in our laboratories have gone on to productive and rewarding research careers, underscoring that this is a formative stage in one’s scientific career. Reflecting on these experiences, through the eyes of trainee and mentor, we hope that this TSR affords some useful tips on whether research is right for you, how to go about procuring a research position, and the broader topic of navigating the LHS/EC stage of your own scientific trajectory.

## Rule 1: Aim for balance: Take your science seriously, don’t take yourself too seriously, and do take others seriously

This Rule is intended in the *opposite* sense of parental rebukes like “*do your homework*,” “*be responsible*,” and so on. Chances are, those pleas don’t apply to those of you reading this piece! Rather, here we implore you to consider that you’re still “a kid” (in a positive sense) and, barring any advances in time travel [2], you won’t be able to return to or relive your teenage years. In short, strive for balance. The rationale for this Rule—and its call for balance—does not lie in pure logic or analytical reasoning, but rather human psychology and the (empirical) principles of social development (e.g., differing levels of various “self-concepts” found among under- and over-achievers [3]). Your deep curiosity about nature, and all the ambitions that stem from that—for the science itself, for your studies and eventual career, and so on—will still exist (in full force) when you’re 21, 25, 30 years old, and beyond. However, the relatively free spirit and less-constrained license of your teenage years generally will *not* be available, at least for most folks; one’s responsibilities (family, job, etc.) grow as a monotonic function through life. Effectively, this Rule is also an argument to enjoy the *journey* versus focusing so single-mindedly on the *destination* that you bypass the other (human and social) opportunities along the way: Those “life experiences” are opportunities for growth of a different kind versus purely technical or scientific. And, by shaping your personality and identity in what are still formative years, your life experiences at this stage will eventually have a far greater impact on you (scientifically, and beyond) than whether or not you can integrate by parts faster than your neighbor. Some of our favorite scientists (who shall remain unnamed) don’t take themselves too seriously, even today; as a concrete example of this Rule manifested, consider the Ig Nobels awarded, for instance, to a science professor who studied why woodpeckers don’t get headaches [4].

This Rule’s advice to “*be principled and high-caliber in your own work*, *and [not ‘but’] also have a healthy respect for other perspectives*” is key throughout your career. We linger on it here because this balance is critical—as an equilibrium between humility (and a healthy respect for others) and self-confidence (enabled by being principled and high caliber in your work). Two tangible examples of these principles at play involve (1) interactions with your advisors; and (2) the peer-review process. As a first example, *taking your science seriously* usually entails engaging with your scientific advisors, from the day-to-day of your work to longer-term matters (e.g., job search). Such engagement will be possible by your being unafraid to ask questions and go out on a limb (*don’t take yourself too seriously*) and knowing that good mentors have stakes both in your short-term success (e.g., a calculation/experiment itself) as well as your long-term aspirations (i.e., your professional success). While being principled, proactive, and ambitious in pursuing your science, also maintain perspective by being humble: Realize that you can learn much from others, at all ages and stages. This leads to our second example—namely, peer review as a chance to practice these principles. In the peer-review process, work submitted to a journal is critiqued, usually by 2 to 4 scientists (“peers”), and the critiques are then relayed to the authors. The key advice is easy: It pays to have a healthy respect for reasonable, evenhanded feedback from a healthy peer-review process (such feedback is how science often progresses, iteratively and incrementally). In all scientific contexts—interactions with advisors and mentors, with journals during peer review, and so on—the greatest benefit comes from balancing humility and a well-founded self-confidence. Strive for this balance from day one, literally: Starting research will likely seem intimidating, but know that you’re not expected to know much, only to be willing to learn. In fact, knowing what you don’t know is the key to progress in any endeavor, not just science. To quote Einstein, “*Imagination is more important than knowledge*. *For knowledge is limited*, *whereas imagination embraces the entire world*…”. All this said, read on!

## Rule 2: Consider what you hope to gain from the experience, but don’t *overthink* the decision—Just do it!

While it is usually beneficial in life to carefully weigh your motivations and decisions, being as honest as possible with yourself, in this case, it may be worthwhile not doing that *too excessively* and just plunging into any reasonable opportunity that becomes available (impulsivity *can* be a plus!). Our rationale here is manifold: (1) Actually *pursuing* a position can help you realize what you don’t like, if the experience isn’t so fun (after 2 to 3 months of committed effort), or it could end up shaping the rest of your life (you discover your calling!). Either way, you’ll learn more about your interests and future paths than when you began. (2) A “just-try-it” mindset can benefit you greatly by avoiding a “paralysis of perfection” that can keep you from pursuing a specific opportunity because it’s not *precisely* the lab/area/locale/etc. that you may have been seeking; balanced against this, don’t just “settle for anything” either, as that could land you in a toxic scientific environment (here, try to trust your intuition and instincts about a lab setting).

Oftentimes, a student will narrow down research opportunities that she’s considering to pursue based on what might “*look good*” or what “*colleges want to see*.” But, at this point in your life, most types of research will look good and will teach you valuable skills; therefore, try to be as open as possible to any reasonable opportunity that presents itself. Do ask yourself if you’re seeking this opportunity for practical (almost “transactional”) purposes, such as being able to list the research experience on your resume? Or, at another extreme, do you simply want to get a taste of hands-on research? As for many things in life, these 2 extremes are *not* mutually exclusive (most of reality sits somewhere between the extremes): Things can be a win-win, whereby you fulfill your scientific curiosity *and* advance your education and career path. Even though it may not feel like it, HS is rather early in your educational trajectory, and if you have any inkling that you may be interested in basic research, now or down the road, then you should just do it. To summarize, we advise (1) keeping an open mind in weighing all reasonable opportunities that arise; (2) exercising judgment in making a final decision; and (3) knowing that, at the end of the day, many decisions in life aren’t as fateful as they may seem in the moment, and overthinking them can backfire.

## Rule 3: Try to take short-, medium-, and long-term views

In terms of your career aspirations and ambitions, ask yourself: How do the positions for which I’m applying “fit in” with my educational, scientific/technical, and career-related goals, on the timescales of approximately 6 to 12 months (short term), 1 to 5 years (medium), and 10+ years (long)? Your ideas in this regard will naturally become more diffuse and much less concrete on the medium and long timescales, but it’s nevertheless worth considering. Also, if you’re very unsure about how your search for a position relates to your short-term plans (e.g., gaining clarity about what you might like to study in college next year), then it’s worth reconsidering aspects of your search process (e.g., should you refocus it on labs that work in areas in which you think you might like to major?). This Rule isn’t meant to present an intimidating challenge (such as mapping out the next decade of your life), nor does it necessarily conflict with Rule 2’s advice to “just do it:” Embarking upon a variety of bite-sized, short-term steps (just trying things) will introduce you to potential mentors, teach you new skills as regards both science and life (e.g., adaptability is key), and ultimately guide you toward defining what could be a satisfying long-term path.

## Rule 4: Cultivate your curiosity and independence

Though it may go without saying, your long-term scientific future (or at least your enjoyment of a scientific path) will ultimately hinge largely upon 2 things: (1) your intrinsic curiosity and passion for Nature; and (2) how much you enjoy thinking and creating for yourself. Independent thinking and curiosity are 2 key pillars of science and research. If you find yourself always asking “Why” and “How” questions, and you sometimes find that pursuing answers leads to your running up against authority figures (parents, teachers, etc.), then you’re probably well motivated for a career in science. Probably in no other field of human endeavor is irreverent thinking as strong an asset as it is in science! (Compelling and oft-humorous examples can be found in the lives of scientific giants like Crick [5], Feynman [6], McClintock [7], and others.) Try not to confuse “*irreverent thinking*” with “*irreverent behavior*”: Seek to be respectful, humane, and gracious toward others, and be sincere in considering their perspectives, technical or otherwise (Rule 1). Try and apply the think-outside-the-box spirit of this Rule not just to your scientific thinking but also to your search for a research position.

## Rule 5: Go ahead and start (before joining a lab): Join scientific mailing lists, get involved in online communities, etc.

Most humans are driven by social instincts and needs and, while they cannot fully substitute for in-person interactions, online communities offer ways both to fulfill those drives and to embark upon scientific research. Today, there’s no shortage of vibrant online scientific communities (see Tip 7 in [8]), from the very general (Quora, Reddit, StackExchange, etc.) to the more specific and technical (GitHub, SourceForge, etc.). Some programming languages have active ecosystems (R is a notable example [9]), as do communities focused at the level of individual software packages (e.g., APBS for biomolecular electrostatics, PyMOL for molecular visualization, the renowned “ccp4bb” in macromolecular crystallography, and so on—each of these have substantial online communities). We mention these resources not to drown you in minutiae, but rather because (1) you may be already involved in some of these digital communities in nonscientific contexts (e.g., Reddit); and (2) the ecosystems that nucleated around certain software suites have, historically, served as quite active and promising venues for advertising positions and career-related opportunities (and, these venues are often unknown to the uninitiated). Similar in spirit, another key resource would be to chat with your science teachers or anyone whom you know to be doing research (personal contacts): *Where did they go to college*? *Might they have any contacts there*, *or other advice*? Similarly, why not email local scientists, in academia and beyond, to ask if you might be able to meet with them for half an hour to discuss their jobs? (Try this with faculty, postdoctoral scientists, graduate students, and upper-level undergraduates.) Also, professional networking sites that are either quite general (e.g., LinkedIn) or more science-specific (e.g., Society for Science & the Public) can afford useful ways to traverse social networks, enabling you to identify “friends of friends” who might be involved in research. The key idea in all this is to try and be as proactive as possible in leveraging the social networking aspects of the internet. Finally, as a practical step, we suggest you consider creating an individual development plan (IDP) as a way to help focus and plan the execution of your educational and career-related activities; though often geared toward those at the graduate and postdoctoral stages, tools such as “myIDP” [10] may be useful earlier on too, such as in your LHS/EC years.

## Rule 6: Think globally (or at least domestically), act locally

By this, we suggest considering all potential research opportunities—apply nationwide, or even internationally—but also focus your enquiries on neighboring research institutions (colleges, universities, national labs, etc.). Admittedly, this is a simpler task in certain metro areas with high densities of research-active labs (United States Bay area, New England, etc.) versus locales where research opportunities may be scarcer; nevertheless, there are often more opportunities than one might initially suspect (e.g., the National Institutes of Health’s Rocky Mountain Laboratories are in a relatively sparsely populated part of the country). On a related note, it doesn’t pay to get caught up on the “name recognition” of institutions: Science is science, and the caliber of it generally won’t vary that much if you’re at an MIT versus a Local Institute of Technology (the *breadth* of potential research fields and available resources will likely differ, but not the quality of an individual research lab/enterprise). To return to the crux of this Rule: The rationale here is partly psychology, partly logistics. First, the psychology: A research group leader, such as a university professor or scientist at a national lab, may be less likely to decline an eager proposal to join their team for a brief stint, e.g., 2 to 3 months over the summer, if you live a short drive away, versus across the country. (Beyond the university setting, note that a group leader may go by various names, for instance, titles like “Senior Investigator,” “Senior Research Associate,” or “Associate Director.”) Second, the logistics: Being physically proximal might present fewer (and lower magnitude) administrative and bureaucratic hurdles versus being remote (e.g., arranging accommodations by the hosting institution, travel/relocation costs, etc.). Logistically, a local institution also makes it easier for *you* to interview your prospective supervisor: You can visit the lab, meet the research advisor and personnel, and get a sense of the lab “culture” [11] and your “fit,” before committing. All of these factors are somewhat intertwined. In contrast, a benefit of visiting a remote lab, e.g., for 10 weeks of intense immersion during summer, is that the experience will almost certainly be life altering, both professionally and personally.

## Rule 7: Try to identify/assess your relative strengths and weaknesses

**Fig 1:**
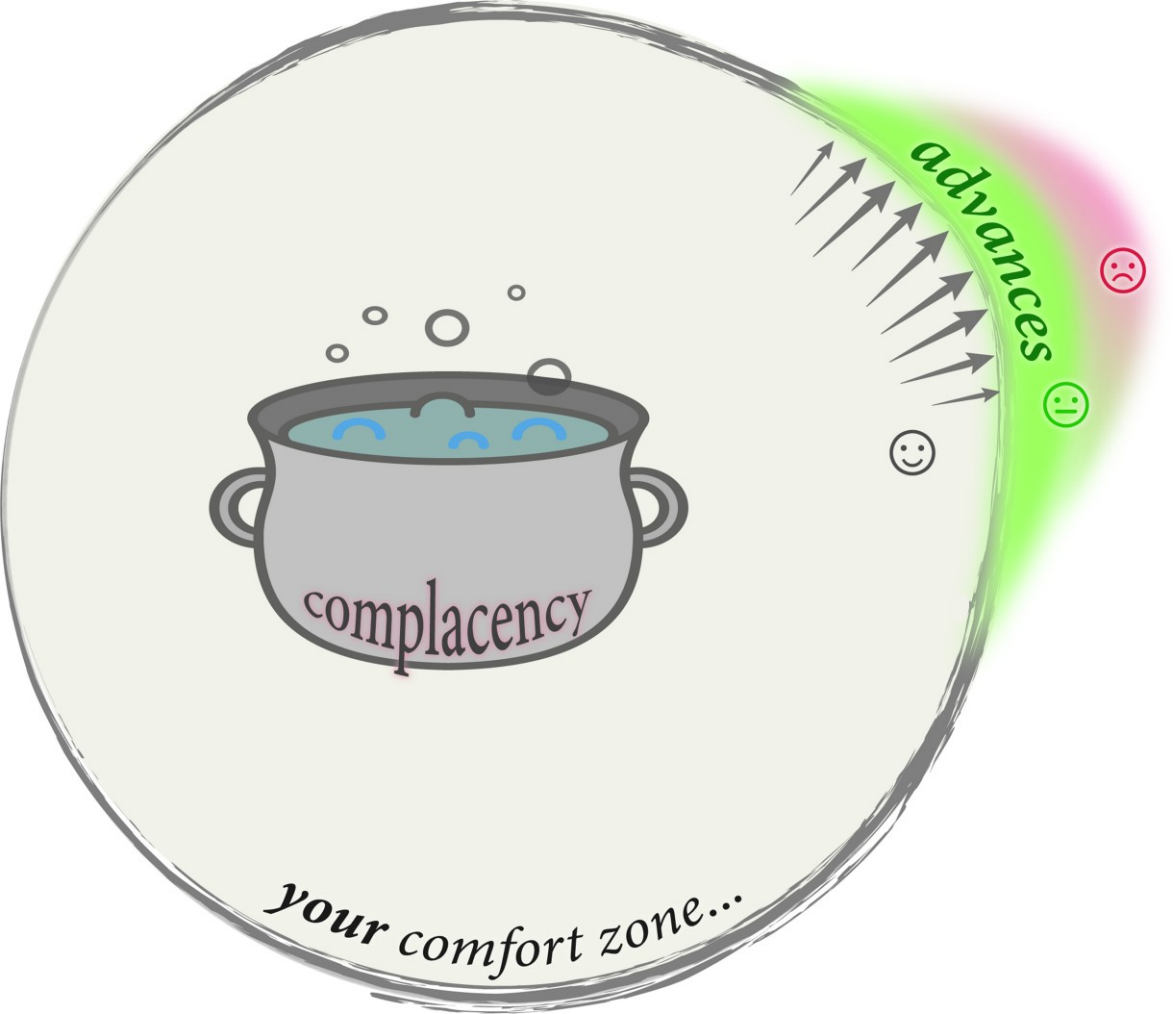
Expand your comfort zone. Advances in anything—learning a new scientific field, a new sport, a new language, how to do research, etc.—occur in a region just beyond the limits of your comfort zone (which really is a *zone* and not an infinitesimally thin line). Pushing your boundaries will take you beyond the realm of complacency, represented by a pot of boiling water (to denote the slowly boiled frog metaphor); doing so gradually and incrementally (via realistic early expectations) across a sweet spot (green zone) will help lessen the frustration (red area) that can arise from jumping too far too fast.

Try to do this as soon as possible (in HS or by the end of your first year of college). This may be difficult. Also, though it may seem frightening, don’t rule out asking others for their takes on your strengths and weaknesses; these can be your peers, teachers, and other/older adults. Take pride in your strengths (and play to your strengths, too) and turn your focus to your potential weaknesses. The reason for this is that you can then begin to address the areas of improvement via coursework, self-study, or whatever mechanisms may be suitable. Some stumbling blocks may take years to work on (e.g., a morbid fear of speaking in front of audiences), so it would be better to start sooner rather than later. As a concrete example, say that you feel your math background isn’t as strong as you’d like; then, you can enroll in more math courses (physically or online). The same goes for writing skills and research skills. In all of this, IDP-like tools (see Rule 5) offer a useful way to help ground, focus, and track your self-improvement efforts. If you’re uncomfortable reading scholarly articles, try using readily accessible tools and platforms (e.g., Google Scholar) to search using a few keywords of interest, and then begin reading a couple articles that seem interesting or compelling to you; initially, we suggest Keshav’s 3-pass approach [12] to reading a paper. This way you’ll begin to glean the general structure of research papers, as well as some of the technical wording. Many other “How-To” guides [13,14] and infographics [15] for reading scientific papers are available, including in the TSR series [16]. (For the inverse activity of *writing* papers, see the many TSRs in Table 1 and a cogent guide by George Whitesides [17].) The fundamental, inescapable principle in all of this is that any sort of growth—personal growth, scientific growth, anything—will occur just outside the boundaries of your personal comfort zone (green region in Fig 1). Always staying inside that zone will engender complacency, while venturing too far beyond it (at once) can set you up for failure and frustration; finding a “sweet spot” and achieving a healthy balance takes practice and is a key (unspoken) element of scientific research.

**Table 1:** From among the 1,000+ Simple Rules that are available [1], the following is an annotated selection of those that might be most helpful at the LHS/EC stage; they are grouped by topic into categories (*Landing a position*, *Making the most* …, etc.).

Title [reference]	Notes, comments
Landing a position	
*TSRs for writing a cover letter to accompany a job application for an academic position* [29]	Though focused on crafting cover letters for academic jobs, many of the same principles apply to any cover letter, for any job (e.g., your letter to potential research mentors).
Making the most of your research position	
*TSRs for providing a meaningful research experience to high school students* [30]	As with most of these TSRs, this piece targets the mentor; that said, there’s no reason you can’t, as a student, peek over at the other side of the fence! At the least, you’ll get a glimpse as to “where your mentor may be coming from” at various times, in various situations. This particular TSR supplies a “*list of programs*, *organized by state*, *that provide high school students with research experiences*;” though not exhaustive or necessarily updated, this list can be an invaluable starting point in your search.
*TSRs for getting the most out of a summer laboratory internship* [31] *TSRs for approaching a new job* [32]	The titles of these pieces speak for themselves: They are invaluable reads when embarking upon your new position.
*TSRs for getting help from online scientific communities* [33]	This useful TSR offers “… *guidelines and suggestions on how to use online communities to solve scientific problems*,” in a manner consistent with good “netiquette.”
*TSRs for building and maintaining a scientific reputation* [34]	Targeted at academic scientists (e.g., lab heads), many of the tips here are useful at all levels. Some of the tips are (hopefully) easier to follow, such as “Never Plagiarize or Doctor Your Data,” while others may require more effort (e.g., “Do Not Ignore Criticism”).
*TSRs for avoiding and resolving conflicts with your colleagues* [35]	This title speaks for itself—the advice is invaluable. For instance, say you experience friction with another researcher in the lab in which you’re working. Among the various ways in which you might respond, are some more productive than others (including not responding at all)?
Communicating science (reading, writing, and presenting) at all levels	
*TSRs for getting published* [36] *TSRs for making good oral presentations* [37]	These 2 TSRs, on writing and on oral presentations, are among the earliest in the series; penned by the founding Editor of this journal, they are foundational with respect to many subsequent TSRs. You’ll likely be asked to give a “group meeting” talk (to your research group) at some point during your work; though relatively informal, you want to do your best at that (see Rule 1).
*TSRs for writing research papers* [38]	Writing papers is “*an essential trait of a… researcher*,” and this TSR advises some writing tips that are intuitive (“*be logical*”), as well as some rules that may be less obvious (e.g., “*be artistic*,” “*consider … referees as your collaborators and treat the reviews with respect*.”)
*TSRs for reading a scientific paper* [16]	This recent TSR offers “*big picture recommendations*,” as well as rules that concern a “*philosophy of reading*” (*be critical*, *kind*, *and invested*) and, finally, tips on “*the ‘now what*?*’ questions… …and how to integrate what was learned into one’s own science*.”
*Ten simple (empirical) rules for writing science* [39]	This TSR appraises the writing tips (rules) that scientists often promulgate (e.g., “*be succinct*”), using empirical data and asking if specific features (e.g., sentence length) correlate with citation frequency in various fields (math, evolution, etc.). Intriguingly, a statistical analysis finds that, “*despite … abundant advice to the contrary*,” more highly cited articles generally feature lengthier prose (at least in the Abstracts).
*TSRs for structuring papers* [40]	This piece, which complements the other writing-related TSRs listed here, offers a systematic and methodical approach to the activity of creating a research article; it describes a C–C–C scheme for structuring a paper as a story that flows in a clear, logical, and compelling manner.
Miscellaneous assortment; general ideas to keep in mind	
*TSRs for doing your best research*, *according to Hamming* [41] *TSRs for lifelong learning*, *according to Hamming* [42]	The gospel according to Hamming: This distillation of some of Hamming’s advice, including his famous “*You and your research*” address (Bells Labs, 1986), offers indispensable advice for researchers at all levels.
*TSRs for online learning* [43]	Though nearly a decade old now, this TSR is still quite salient—particularly during a pandemic, when many classes have gone online-only, you may wish to begin learning a new field or skill set (e.g., programming, per Rule 7 in [44]), and so on.
*TSRs for teaching Bioinformatics at the high school level* [45]	Here, we can substitute “*teaching*” → “*learning*,” as the same advice applies: Teaching and learning are 2 sides of the same coin, and this TSR shows you the coin.
*TSRs for effective statistical practice* [46]	This compilation of advice on applied statistics is useful at all levels, HS and beyond.
*TSRs for biologists learning to program* [20]	A valuable primer for those new to programming. See also related (and complementary) offerings, such as *A Quick Guide to Organizing Computational Biology Projects* [47] and *An Introduction to Programming for Bioscientists*: *A Python-Based Primer* [19].
*TSRs to make the most out of your undergraduate research career* [48]	The title of this TSRs could just as well have ended as “. . .*high school research career*”: The advice and general principles apply there too.
*TSRs for graduate students* [49]	While some of this is grad school specific (e.g., Rule 10 about the thesis committee), much of it also applies at any level, LHS/EC and beyond.
*TSRs towards healthier research labs* [11]	Again, many of the Rules articulated in this piece apply at all levels, from LHS/EC onward (e.g., the advice to “*Destigmatize failure and celebrate success*”).
*TSRs for protecting research integrity* [50]	This TSR, covering ethical principles and scientific misconduct, applies at all career stages, from HS to retirement: It supplies advice, reminders, and summaries of best practices at the levels of both individuals (e.g., coauthorship practices) and institutions (e.g., whistleblower policies).
*TSRs for getting involved in your scientific community* [51]	A TSR full of useful advice and reminders—e.g., “*Jump into the Pool and Get Involved*,” “*Work in a Team*,” and so on.

C–C–C, context–content–conclusion; HS, high school; LHS/EC, late high school/early college; TSR, Ten Simple Rules.

## Rule 8: Take some coding and stats classes

Programming and statistics are 2 of the most indispensable areas in modern science, and they also underpin “hot” fields such as data science [18]. Taking this a step further, you could even argue that any job of the future will likely require some computer programming and statistical analysis skills. Thus, at least some training in these areas will position you well in practical terms: Beyond science-in-academia, if you eventually find yourself gravitating toward industry or other sectors, a minimal background in software engineering, data analytics, machine learning, etc., will make you highly marketable. This advice is being singled out as a Rule because the curricula for many college degree programs, even in the physical sciences (e.g., chemistry), do not require any statistics or computer science coursework; we urge you to pursue courses in these subjects, irrespective of formal degree requirements. For an autodidactic approach, many online platforms are available for self-teaching (e.g., Codecademy, freeCodeCamp, code.org), as well as freely accessible, self-contained primers on popular languages such as Python [19,20]. Numerous open educational resources, including massive open online courses (MOOCs), are cataloged at Wikipedia; in particular, the category class “open educational resources” [21] provides a portal of (actively updated) links to such resources as Coursera, edX, Khan Academy, Udacity, and so on.

## Rule 9: Flexibility pays (especially in the short run), and persistence pays too (especially in the long run)

If you don’t land exactly the position you’d hoped to—but you have another offer that’s at least minimally acceptable (even if it differs at the level of being in a quite different scientific field from what you’d originally sought out)—that’s fine, take it! Why? Because, while persistence and even stubbornness will serve you well in your ultimate commitment to being a scientist/researcher, it pays to be flexible as regards the opportunities and paths that get you there. Keeping an open, adaptable mindset about even seemingly large-scale issues is beneficial in the long run, and the flexibility that it fosters also can be greatly helpful on shorter timescales: In performing research, we draw upon our cognitive flexibility relentlessly, even if often unknowingly. For example, to troubleshoot code or a failed experiment, one often begins by imagining and considering as many viable alternative scenarios as possible, even if they may seem mutually exclusive (holding multiple viewpoints and entertaining dissonant ideas is a virtue in scientific and creative thinking). It’s helpful to bring a degree of flexibility to your immediate search for a position. Longer term, a well-grounded stubbornness likely will aid your scientific career; note that we include the qualifier “well-grounded” because flexibility and openness to new options, alternative paths, etc., are key, too (in the same spirit as the short-term component of this Rule). In practice (and with practice), you’ll eventually develop a feel for when it pays to be more versus less stubborn. The greater balance you can achieve between these—flexibility/adaptability and stubbornness/persistence—the more likely you are to be successful and content with your career path, in the long run.

## Rule 10: Mitigate multitasking

At all stages, from HS to beyond, you will ultimately benefit from limiting multitasking. When listening in a brick-and-mortar classroom or online, thinking in a library, pipetting at a wet lab bench, compiling code in Linux—in short, in any form of learning—try to minimize multitasking. (Maybe set aside daily times for activities that demand less focus, as suggested at the end of Rule 5 in [22]?) We preach this here because chronic multitasking, e.g., an “always-on” approach to email, social media, etc., is now understood to impair one’s cognitive health and ability to focus: The addiction and brain chemistry aspects of these phenomena have been reported in popular articles long ago [23], peer-reviewed research articles [24,25,26], and whole books on the topic [27]. To really *learn* and *do* science require periods of intense focus. (Though, intriguingly, a *perception* of multitasking can actually enhance performance [28].) The same sermon applies to texts, blogs, feeds, streams, and so on: bad for your health and (maybe) your science. We realize that this Rule runs counter to much of what is culturally popular (societal norms, ca 2020), including even that which appears in this journal [22]. That’s fine: Differing perspectives and disagreement are part and parcel of science! Rather than be irascible or backward about it, we simply implore you to beware the addictive capacity of social media, given its potentially deleterious cognitive effects.

## Conclusions

In closing, we believe, biased though we are, that a career in research is a great way to spend one’s professional life. Is it for you? The only way to definitively find out is to simply try it. In what stretches before you, what have you got to lose? If you’re unsure, perhaps examine some of the other articles in the TSR series to help you decide; Table 1 provides an annotated list of TSRs that could be most helpful as you begin your journey.
